# MVBNSleepNet: A Multi-View Brain Network-Based Convolutional Neural Network for Neonatal Sleep Staging

**DOI:** 10.1109/OJEMB.2025.3548002

**Published:** 2025-03-05

**Authors:** Ligang Zhou, Minghui Liu, Xia Hu, Laishuan Wang, Yan Xu, Chen Chen, Wei Chen

**Affiliations:** School of Information Science and TechnologyFudan University 12478 Shanghai 200433 China; Human Phenome InstituteFudan University 12478 Shanghai 201203 China; Department of NeonatologyChildren's Hospital of Fudan University, National Children's Medical Center 145601 Shanghai 201102 China; Center for Medical Research and InnovationShanghai Pudong Hospital 542170 Shanghai 201203 China; Human Phenome InstituteFudan University 12478 Shanghai 201203 China; School of Biomedical EngineeringUniversity of Sydney 4334 Sydney NSW 2006 Australia

**Keywords:** Brain network, deep learning, EEG, functional connection, neonatal sleep staging

## Abstract

*Goal:* To develop a high-performance and robust solution for neonatal sleep staging that incorporates spatial topological information and functional connectivity of the brain, which are often overlooked in existing approaches. *Methods:* We propose MVBNSleepNet, a multi-view brain network-based convolutional neural network. The framework integrates a multi-view brain network (MVBN) to characterize brain functional connectivity from linear temporal correlation, information-theoretic, and phase-dynamics perspectives, providing comprehensive spatial topological information. A masking mechanism is employed to enhance model robustness by simulating random dropout or low-quality signal conditions. Additionally, an attention mechanism focuses on key regions of the brain network and reveals structural brain connectivity during sleep, while a CNN module extracts spatial features from brain networks and classifies them into specific sleep stages. The model was validated on a clinical dataset of 64 neonatal EEG recordings using a leave-one-subject-out validation strategy. *Results:* MVBNSleepNet achieved an accuracy of 83.9% in the two-stage sleep task (sleep and wakefulness) and 76.4% in the three-stage task (active sleep, quiet sleep, and wakefulness), outperforming state-of-the-art methods. *Conclusions:* The proposed MVBNSleepNet provides a robust and accurate solution for neonatal sleep staging and offers valuable insights into the functional connectivity of the early neural system.

## I. Introduction

Sleep is essential for the development of the brain, cognitive, and early sensory system of the neonate [1]. To evaluate the quality or structure of the neonate's sleep, sleep staging is regarded as the basic and essential procedure, which can reveal the construction and mechanism of sleep. Polysomnography (PSG) is considered the gold standard for sleep analysis, in which sleep staging is normally performed by trained physicians by recognizing the characteristics of PSG visually. However, it is monotonous and time-consuming work, and subjective interpretation exists among physicians. Thus, numerous automatic sleep staging methods are proposed to alleviate the physician's workload and to offer a relatively objective evaluation of sleep staging.

Currently, deep learning-based methods for automatic sleep staging have sprung up, which are almost prior-knowledge-free and achieve favorable performance. These methods can be roughly categorized into limited-channel and multi-channel EEG-based approaches. Regarding limited-channel EEG-based approaches, Ansari et al. proposed an end-to-end deep learning architecture for automated Quiet Sleep (QS) detection, in which a variation of the Convolutional Neural Network Inception block named Sinc is adopted [2]. Afterward, Zhu et al. proposed a multi-scale hierarchical neural network (MS-HNN) with a squeeze and excitation (SE) block for three stages (namely, Wakefulness, Active Sleep (AS), and QS) classification [3]. Although single/limited channel EEG-based approaches achieved favorable performance in sleep staging, potential information loss and performance degeneration may exist when it comes to the model's generalizability, spatial information, and interference with the signal. Therefore, some studies have moved the focus on multi-channel EEG-based approaches, such as Ghimatgar et al. combined hand-crafted feature extraction, feature selection, bi-directional long-short-term memory (Bi-LSTM), and HMM optimization for AS, QS, and their sub-states classification [4]. While simply increasing the number of EEG channels would provide supplemental information, it may also contribute to redundant information. Besides, for the existing multi-channel EEG-based approaches, spatial topological information and functional connectivity of the brain during sleep have been ignored.

Several studies have explored the feasibility of spatial topological information in sleep staging. Stevner et al. demonstrated the dynamic choreography between different whole-brain networks across the wake-non-REM sleep cycle, which reveals key trajectories to switch within and between EEG-based sleep [5]. Also, graph neural networks (GNN) are used in sleep staging, such as Jia et al. proposed an adaptive spatial-temporal graph convolutional network for sleep staging named ‘GraphSleepNet’ for adult sleep staging by performing graph convolution following an adaptive graph learning method they introduced [6]. Afterward, a multi-view spatial-temporal GCN with domain generalization for sleep staging is proposed to effectively extract the features from both spatial and temporal domains and overcome individual variance [7]. Meanwhile, Huang et al. have applied phase-locked value (PLV) to build the functional connectivity network with the utilization of the information between EEG channels and brain areas for sleep staging [8]. Whereas, simply applying these adult sleep staging methods to neonatal sleep staging is not appropriate, because the sleep micro-events, sleep dynamics, sleep structure, etc. are not consistent [9], [10], [11], [12]. In addition, simply increasing the number of channels without considering the relationship between channels, or only involving a single view of the relationship ignores the structure of these connectivity from different perspectives.

To address these challenges, it is imperative to harness the wealth of information embedded in multi-channel EEG data while considering the intricate relationships in multi-view brain networks. A multi-view brain network-based convolutional neural network (MVBN) is proposed. To characterize spatial topological information of the brain, it assesses the functional connectivity from various perspectives including linear temporal correlation, information-theoretic, and phase-dynamics perspectives. Subsequently, the Hadamard product is computed by element-wise multiplication of a set of trainable weight matrices and the derived brain networks. This composite feature is then inputted into the Convolutional Neural Network (CNN) model. The learned weight matrices serve to unveil the significance of connections within the brain networks, with higher weight values signifying increased relevance to both connections and sleep stages. The main contribution of this study can be summarized as follows:

1) Brain functional connectivities are characterized using topological features of PCC, MI, and phase-related metrics from linear temporal correlation, information-theoretic, and phase-dynamics perspectives. This approach provides a comprehensive view of brain connectivity and a novel perspective on sleep staging.
2) A data-driven brain network is proposed for neonatal sleep staging, aiming to yield interpretable outcomes in the context of neonatal sleep classification.
3) A masking mechanism is introduced to mimic random signal dropout or low-quality signals, assessing the model's robustness and minimum channel requirements. It also evaluates the model's adaptability to diverse EEG channel configurations.

## II. Materials and Methods

### A. Experimental Data and Preprocessing

The sleep EEG data we used was recorded from 64 neonates of 38-43 weeks postmenstrual age, who suffered from some diseases such as bloating, hyperbilirubinemia, jaundice, pneumonia, and so on, at Children's Hospital of Fudan University in 2017-2018. The data collection for this study was approved by the ethics committee of the CHFU (approval No. (2017) 89). In total, 64 sleep EEG recordings with an average data length of 131 min were collected by a Nicolet device with the EEG channels of F3, F4, C3, C4, T3, T4, P3, and P4 at a sampling rate of 500 Hz according to the international 10-20 system of electrode placement. The annotation of the sleep staging was visually performed by the neurophysiologist, who identified the sleep into three stages wakefulness, Quiet sleep (QS), and Active sleep (AS) based on the physiological recordings. Considering the practical guidelines and recommendations for neonatal sleep staging, the EEG signals were partitioned into epochs with a length of 30 seconds. The specifications of the neonates and the dataset are provided in Table I.

**TABLE I:** Specifications of the CHFU Dataset

Terms	Details
Gender (b: g)	32:32
Gestational age (w + d)	38.3 $\pm$ 1.8
Postmenstrual age (w + d)	40.5 $\pm$ 1.7
Weight (kg)	3.3 $\pm$ 0.6
Number of W epochs	5514
Number of QS epochs	5749
Number of AS epochs	5540
Reason for Inclusion	Pneumonia, jaundice, etc.
EEG channels	8
Sampling rate	500 Hz

^0^*b: g means boy: girl and w + d denotes week + day.

To mitigate noise and address powerline frequency interference, a notch filter centered at 50 Hz and a band-pass filter ranging from 0.3 Hz to 35 Hz are implemented on the EEG signals. For filtering, we employed a zero-phase 5th-order Butterworth band-pass filter, chosen for its ability to effectively attenuate noise and unwanted frequencies while preserving signal quality. Epochs containing interruptions, man-made interference, and questionable signals are excluded from subsequent processing. Additionally, the EEG signals are down-sampled from 500 Hz to 100 Hz to optimize computational efficiency in further analysis.

### B. Proposed Method

**Fig. 1.**
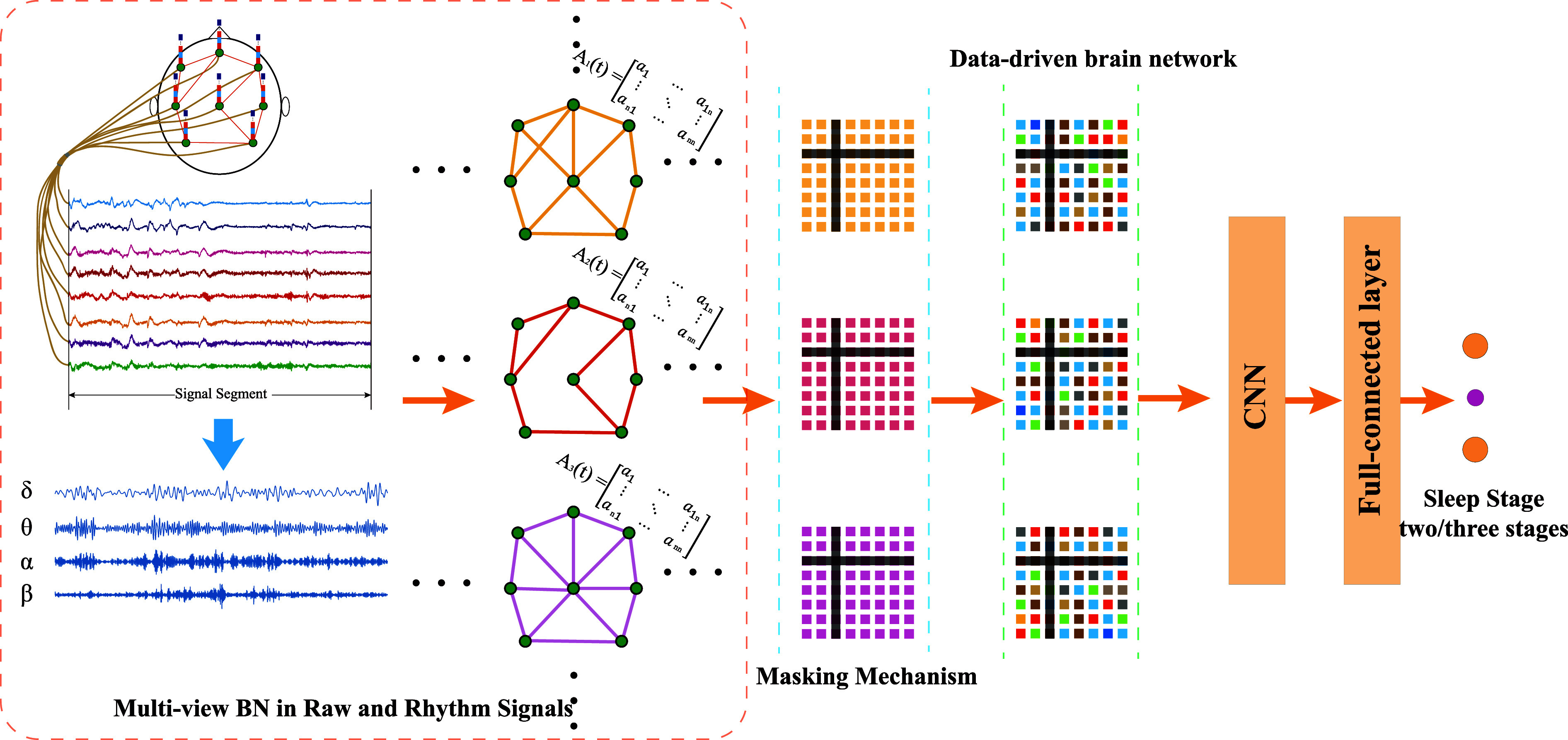
Overall structure of the method.

The architecture of the proposed MVBNSleepNet is shown in Fig. 1, which consists of sub-frequency band signals extraction, brain network extractor, masking mechanism, data-driven mechanism, CNN model, and fully connected layers. In the model training phase, a cross-entropy loss function and an Adam optimizer were applied to update the parameters of the model.

#### 1) Establishment of Multi-View Brain Network in Raw and Rhythmic Signals

Sleep stages are recognized visually by neurologists based on the characteristics of the PSG signal. The distribution of sub-frequency signals (rhythmic wave: $\delta$, $\theta$, $\alpha$, and $\beta$) is an indispensable component, which is used by neurologists for sleep staging. To harness the distinctive features inherent in sub-frequency signals, rhythmic waves ($\delta$, $\theta$, $\alpha$, and $\beta$) are extracted using zero-phase fifth-order Butterworth bandpass filters with pass-bands set at 1–4 Hz, 4–8 Hz, 8–13 Hz, and 13–30 Hz, respectively [13]. In this study, the rhythmic waves were extracted for all subjects utilizing 8 EEG channels.

These functional connectivity measures—PCC, MI, PLV, PSI, and PLI—were selected due to their capacity to capture distinct dimensions of brain dynamics critical for neonatal sleep staging. PCC quantifies the linear correlations between EEG channels, reflecting levels of synchronous neural activity that vary by sleep stage. MI further extends this by capturing both linear and nonlinear dependencies, thereby offering a more comprehensive view of the complex interactions within the developing neonatal brain. For the phase-related information, to address volume conduction and phase distortion in phase-related information, we utilized multiple phase-based connectivity measures, each offering unique advantages, which also can be complemented by the Mutual Information (MI) and Pearson Correlation Coefficient (PCC). PLV measures the consistency of phase differences across signals, which is crucial for characterizing oscillatory brain behavior across sleep stages. PSI and PLI, by focusing on directional and non-directional phase relationships, respectively, provide insight into the consistency and directionality of phase coupling, aiding in the detection of subtle connectivity changes associated with different sleep stages and developmental maturation. Collectively, these connectivity measures afford a multidimensional perspective on brain network dynamics, enhancing the robustness and accuracy of neonatal sleep staging. This analysis is conducted on both the original EEG signal and signals derived from brain rhythms. The elements of $A_{pcc}$, $A_{mi}$, $A_{plv}$, $A_{pli}$, and $A_{psi}$ are calculated as follows:

\begin{align*}
a^{ij}_{pcc}=& \frac{\text{cov}(c_{i}, c_{j})}{\sqrt{\text{var}(c_{i}) \cdot \text{var}(c_{j})}}. \tag{1}\\
a^{ij}_{mi}=&\sum _{c_{i}} \sum _{c_{j}} p(c_{i}, c_{j}) \log \left(\frac{p(c_{i}, c_{j})}{p(c_{i})p(c_{j})}\right). \tag{2}\\
a^{ij}_{plv}=&\left| \frac{1}{N} \sum _{t=1}^{N} e^{i\Delta \phi (t)} \right|. \tag{3}\\
a^{ij}_{pli}=&\left| \frac{1}{N} \sum _{t=1}^{N} \text{sign}(\Delta \phi (t)) \right|. \tag{4}\\
a^{ij}_{psi}=&\frac{1}{N-1} \sum _{t=1}^{N-1} \left| \Delta \phi (t+1) - \Delta \phi (t) \right|. \tag{5}
\end{align*}

where $c_{i}$ and $c_{j}$ denote the EEG channel $i$ and channel $j$ and $a^{ij}$ denotes the element of matrix A with the location of $(i, j)$. $\Delta \phi (t)$ refers to the phase difference between between EEG channel $i$ and channel $j$ at time point $t$.

The raw EEG signals sequence can be defined as $E=(e_{1}, e_{2},{\ldots }, e_{L}) \in \Re ^{L \times C \times S}$, where $L$ is the length of sleep epochs, $C$ means the number of EEG channels and the $S$ denotes the samples of one channel in each epoch. In this study, the raw EEG signals are divided into 30s time lengths with a sampling rate of 100Hz. For each sleep epoch $e_{i}$, a series of brain networks are extracted by the method mentioned above, which constitute a multi-view brain network $M=(m_{1}, m_{2},{\ldots }, m_{N}) \in \Re ^{N \times C \times C}$, where $N$ is the number of brain networks and the $C$ represents the number of EEG signals channel. Finally, a sequence of brain networks of each epoch will be obtained, which can be represented as $G=(M_{1}, M_{2},{\ldots }, M_{L}) \in \Re ^{L \times N \times C \times C}$.

#### 2) Data-Driven Brain Network

After the extraction of the brain network, an attention mechanism between different brain networks is proposed to generate a towards-task brain network by the data-driven method in specific tasks such as sleep staging. In the proposed data-driven method, a set of trainable weight matrices $W=(w_{1}, w_{2},{\ldots }, W_{N}) \in \Re ^{N \times C \times C}$ are used to learn the specific weight in the task through a neural network layer. Finally, the learned data-driven brain networks $G^{D}=(M_{1}^{D}, M_{2}^{D},{\ldots }, M_{L}^{D}) \in \Re ^{L \times N \times C \times C}$, in which each learned brain network $m^{D} \in \Re ^{C \times C}$ in one sleep epoch is defined as:

\begin{equation*}
m^{T}=f(e)=Softmax(Relu(w^{D} \circ g(e))). \tag{6}
\end{equation*}

where the $g$ is the function for extracting the brain network, $\circ$ means the Hadamard product operation, $w^{D}$ denotes the learned weight of a single brain network view, and a rectified linear unit (ReLU) function is used to guarantee the positive definiteness of the Hadamard product. Finally, a view level normalization is conducted using the softmax operation. The trainable weight matrices are updated with the model training by minimizing the system loss function.

#### 3) CNN and Fully Connected Layers

A lightweight CNN is designed for extracting the features of brain networks, which consist of 10 matrixes with a size of $8*8$. The kernel size of the first two CNN layers is $3*3$, and a kernel with the size of $2*2$ for the last CNN layer. Each CNN layer is followed by a Rectified Linear Unit (ReLU) activation layer. After the feature extraction by three CNN layers, we employ four linear layers to learn the weight of features and output the logits of sleep stages. The output shape of each layer is shown in Table II.

**TABLE II:** The Output Shape of Each Layer

Step	Output
Raw EEG signal	[batch, 1, 30*sr]
Rhythm extraction	[batch, 10, 30*sr]
Brain network extraction	[batch, 10, 8, 8]
CNN layer 1	[batch, 16, 6, 6]
ReLU layer 1	[batch, 16, 6, 6]
CNN layer 2	[batch, 32, 4, 4]
ReLU layer 2	[batch, 32, 4, 4]
CNN layer 3	[batch, 64, 3, 3]
ReLU layer 3	[batch, 64, 3, 3]
Flatten layer	[batch, 1152]
Linear layer 1	[batch, 576]
Linear layer 2	[batch, 256]
Linear layer 3	[batch, 128]
Linear layer 4	[batch, 64]
Linear layer 5	[batch, 3]

^0^*sr denotes the sampling rate of the EEG.

### C. Masking Mechanism

**Fig. 2.**
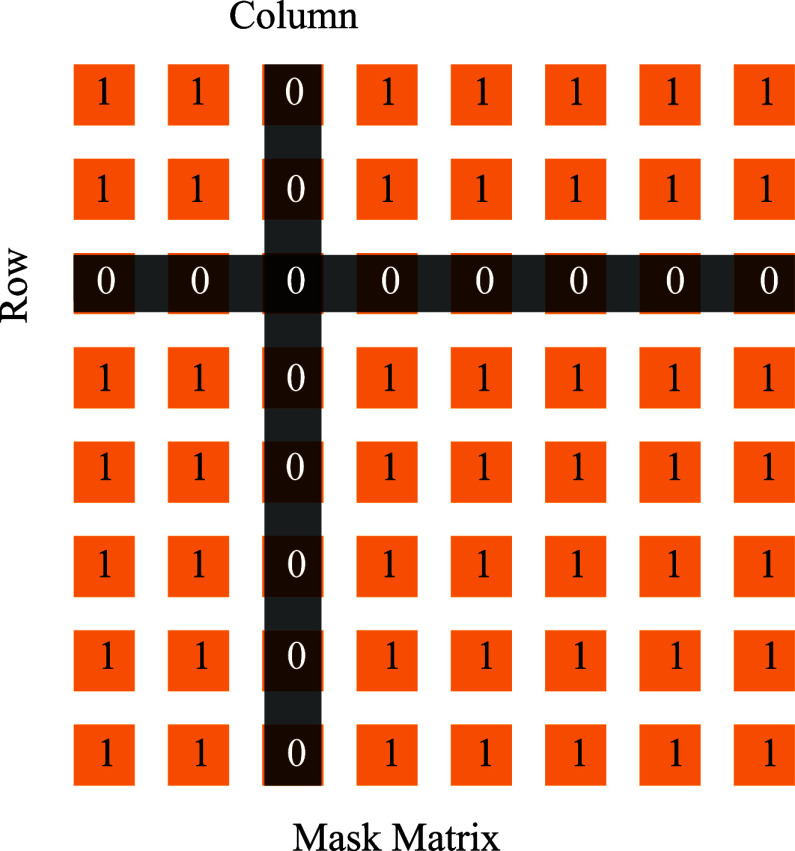
Masking mechanism: the mask is generated by setting the specific locations as zero in an all-one matrix.

To evaluate the model's robustness at the channel scale and align it more closely with real-world experimental conditions, we introduce a masking mechanism before the CNN layers. This mechanism simulates scenarios such as electrode shedding or signal loss during actual signal collection experiments. To simplify the implementation and maintain focus on the core methodology, we opted to simulate poor-quality signals by directly masking the corresponding rows and columns of the connectivity matrix. This approach effectively achieves the same goal while reducing computational complexity and keeping the analysis tractable. The masking process involves the utilization of a matrix, treated as the mask, generated by introducing zeros at specific locations within an all-one matrix, as illustrated in Fig. 2. Models incorporating the masking mechanism entail an additional step, wherein the mask is applied through bit-wise multiplication to the brain networks along the view axis. This process can be articulated as follows:

\begin{equation*}
A_{masked}=M*A. \tag{7}
\end{equation*}

where, $M$ indicates the mask matrix, $A$ denotes the original brain network and $A_{masked}$ is the masked brain network.

### D. Evaluation Metrics

The Leave-One-Subject-Out (LOSO) method is implemented to assess the performance of the proposed methodology across diverse subjects. Performance metrics, such as accuracy (ACC), F1-score, kappa coefficient, specificity, and sensitivity, are employed for evaluation and comparative analysis with other methods. Additionally, precision for each class is considered. The training and testing of the model are executed in Python on a personal computer with 16 GB RAM and a 2.9 GHz i5 processor.

## III. Results

### A. Performance Evaluation

**Fig. 3.**
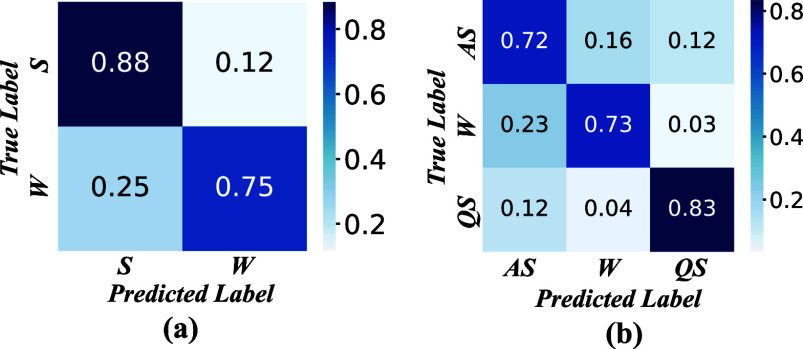
Performance of the proposed model. (a) Normalized confusion matrix of the model on two-stage task. (b) Normalized confusion matrix of the model on three-stage task.

The proposed method undergoes comprehensive evaluations in both two-stage and three-stage tasks, employing the Leave-One-Subject-Out (LOSO) validation method, as illustrated in Fig. 3. In the two-stage task, distinguishing between sleep and wakefulness, the proposed method achieves notable performance metrics: an accuracy of 0.839, an F1-score of 0.817, a kappa coefficient of 0.634, a specificity of 0.816, and a sensitivity of 0.816. Precision values are reported as 0.88 and 0.75 for sleep and wakefulness, respectively. For the three-stage task involving active sleep (AS), wakefulness, and quiet sleep (QS), the proposed method demonstrates commendable results: an accuracy of 0.764, an F1-score of 0.764, a kappa coefficient of 0.645, a specificity of 0.882, and a sensitivity of 0.763. Precision values are reported as 0.72, 0.73, and 0.83 for AS, wakefulness, and QS, respectively.

### B. Comparison With State-of-Art Methods

In this study, we conducted a comparative analysis between our proposed method and state-of-the-art approaches. The evaluation was performed on our clinical dataset from a prior study, wherein the training iteration was set to 150 iterations, taking into account early stopping criteria for certain methods [3]. All state-of-the-art methods employed a single-channel EEG signal (P4) as input, aligning with their original architectures that produce outputs for three sleep stages. In contrast, our proposed method utilized eight EEG channels as input with an output corresponding to three sleep stages. Furthermore, each method underwent equal evaluation through the subject-wise 10-fold cross-validation method, which accounts for both the diversity of the data and the variability between subjects and allows for more comprehensive data utilization to train the model effectively, while also reflecting the model's robustness across different subjects. The specific details of these state-of-the-art methods are outlined below:

The detailed performance results of these methods are presented in Table III. It is evident from the outcomes that the proposed method consistently outperforms the state-of-the-art methods. Notably, the Conv-2 d method [14], [15] encounters challenges in adequately extracting information from the signal, leading to underfitting issues. To explore the feasibility of transferring adult sleep models to neonatal sleep data, the adult sleep staging models DeepSleepNet [16] and AttnSleep [17] were included in this experiment. The results reveal a significant performance decrease in neonatal sleep data, underscoring the dissimilarities in sleep features between neonates and adults. When directly applied to neonatal data, GraphSleepNet [6] and MVST-GCN [7], which are specifically designed for adult sleep staging with multi-channel EEG, show significantly reduced performance: GraphSleepNet achieves an overall accuracy of 0.689, F1 score of 0.682, the sensitivity of 0.535, specificity of 0.845, and kappa of 0.692, while MVST-GCN achieves an overall accuracy of 0.697, F1 score of 0.696, the sensitivity of 0.547, specificity of 0.849, and kappa of 0.699. In contrast, our proposed method, which is designed to better accommodate neonatal EEG characteristics, achieves higher results across all metrics with an overall accuracy of 0.797, F1 score of 0.797, sensitivity of 0.697, specificity of 0.901, and kappa of 0.802. These results demonstrate that our approach is more suitable for neonatal EEG data, achieving superior performance compared to models optimized for multi-channel adult EEG, thus highlighting its robustness and adaptability for neonatal sleep staging.

**TABLE III:** Performance Comparison of State-of-Art Methods and the Proposed Method

Methods	Acc	F1-score	Kappa	Spe	Sen
Conv-2d [14]	0.535	0.531	0.489	0.768	0.536
Conv-2d [15]	0.523	0.519	0.411	0.761	0.523
DeepSleepNet [16]	0.698	0.689	0.644	0.805	0.703
AttnSleep [17]	0.680	0.646	0.659	0.839	0.650
THNN [18]	0.694	0.686	0.638	0.848	0.698
MS-HNN [3]	0.754	0.758	0.728	0.876	0.755
GraphSleepNet [6]	0.689	0.682	0.535	0.845	0.692
MVST-GCN [7]	0.697	0.696	0.547	0.849	0.699
**Proposed Method**	**0.797**	**0.797**	**0.697**	**0.901**	**0.802**

## IV. Discussion

Considering data quality inconsistencies across sources, we introduced a masking mechanism to simulate poor-quality data. Experimental results confirm our method's resilience under such conditions. However, limitations exist. First, poor-quality signals were simulated by masking rows and columns of the connectivity matrix, not the input signals, which may not fully replicate real conditions. Future work should investigate input-level signal quality impacts to enhance robustness. This study provides a preliminary evaluation of performance in these scenarios, warranting further research to mitigate poor data impacts and explore robustness across populations. The neonatal EEG data used were from clinical settings, excluding psychiatric disorders and psychotropic medication use, reflecting real-world conditions while avoiding confounding factors. Second, while our approach leverages functional and effective connectivity for automated sleep staging, it does not fully explain sleep's physiological or structural mechanisms. Future studies could integrate multimodal data and advanced techniques to deepen insights into sleep processes. These limitations underscore areas for improvement and further validation of our method.

## V. Conclusion

A multi-view brain network-based CNN method is proposed for neonatal sleep staging using multi-channel EEG. By incorporating PCC, MI, and phase-related networks, it extracts spatial topological and functional connectivity features, reducing data volume while intuitively linking brain states to sleep. The method demonstrates high performance and robust generalization to both neonate and adult sleep datasets, supporting broader clinical and research applications. A masking mechanism enhances adaptability to heterogeneous data, addressing signal inconsistencies. This approach reduces medical workload and extends advanced sleep analysis technologies, benefiting both pediatric and adult populations in sleep and brain science advancements.

## Supplementary Materials

The supplementary material provides comprehensive details to support the main study, including the basics of brain networks, an illustration of brain network establishment, and the data-driven brain network framework. It includes ablation experiments analyzing the effects of different brain network scenarios and masking mechanisms, along with detailed comparisons with state-of-the-art methods. Additionally, a supplementary discussion explores key aspects, such as the impact of single-view brain networks, EEG channel variations, and the generalization of our method to adult sleep datasets. Readers are encouraged to refer to the supplementary material for in-depth insights and extended analyses.

## Ethics Statement

The data collection for this study was approved by the ethics committee of the CHFU, under the approval number (2017) 89. All participants provided written informed consent prior to their involvement in the study, and the research was conducted in accordance with the Declaration of Helsinki.

## Conflict of Interest Statement

The authors declare that there are no conflicts of interest associated with this study or its publication.

## Author Contributions Statement

Ligang Zhou and Minghui Liu contributed to the study's conception, experimental implementation, result organization, manuscript drafting, and critical revision. Xia Hu participated in the proofreading and review of the manuscript. Laishuan Wang and Yan Xu provided data support and contributed to the manuscript review and proofreading. Chen Chen and Wei Chen provided methodological insights, funding support, and participated in the manuscript's review and refinement. All authors reviewed and approved the final version of the manuscript for submission.
